# Study of the effect of bacterial-mediated legume plant growth using bacterial strain *Serratia marcescens* N1.14 X-45

**DOI:** 10.3389/fmicb.2022.988692

**Published:** 2022-10-24

**Authors:** Jiaxin Zheng, Chao Liu, Jiayi Liu, Jia Yao Zhuang

**Affiliations:** Collaborative Innovation Center of Sustainable Forestry in Southern China of Jiangsu Province, Nanjing Forestry University, Nanjing, China

**Keywords:** bacterial communities, bioremediation, phytoremediation, sequencing technology, soil fertility

## Abstract

Soil microorganisms play an indispensable role in plant growth and are widely used to promote plant growth. However, poor microbial strains are homogeneous. The heavy application of chemical fertilizers and pesticides to agricultural soil has adversely affected the soil flora, necessitating the regulation of the soil flora to maintain soil health. In this study, X-45, a highly efficient and phosphorus-dissolving strain of the lysogenic bacterium *Serratia marcescens* N1.14 was isolated from bare rock slope soil samples from Yueyang Avenue, Hunan Province, China. We observed that microbial strain X-45 could release P from the rocks into solution when the sample rocks were used as the only phosphorus source. Furthermore, we observed that the P content in media increased by 3.08 X compared to the control. After applying X-45 as a bacterial fertilizer, the growth of potted *Indigofera pseudotinctoria* plants significantly increased, the soil physicochemical properties were significantly improved, and the relative abundance of *Bradyrhizobium* in the soil increased significantly from 1 to 42%. Besides, *Bradyrhizobium* became the most dominant genus in the soil. The indirect promotion of another beneficial microorganism by X-45 further revealed the intrinsic mechanism by which X-45 exerted its effect on plant promotion and soil improvement. Using this bacteria, the hypothesis of the superposition effect of legume plant promotion was also confirmed.

## Introduction

The restoration of high and steep exposed rock walls demands urgent attention as human activities, such as road construction and mining, have led to the exposure of high and steep slopes, adversely affecting the ecological habitat of human beings. Phytoremediation is considered the most promising restoration technology because of its low cost and environmental friendliness. However, restoration of exposed rock walls is challenging because they are mainly made of hard rocks, and their poor ground conditions make it difficult for plants to grow. The improvement of environmental conditions is crucial to the restoration of exposed high and steep slopes. Soil microorganisms have a vital role in rock weathering (Zhu et al., 2014). Besides, soil microbes enhance the release of rock nutrients and promote plant growth (Wu et al., 2017). For instance, bacteria actively solubilize phosphorus, which is then transported by mycorrhizal fungi to the plant root system (Song et al., 2021). Soil microbes also facilitate rock dissolution, creating a microenvironment conducive to plant growth (Mo and Lian, 2011). The weathering rate of minerals and rocks by microbial activities is significantly higher than in other abiotic systems (Barker et al., 2018). For instance, microorganisms play a positive role in CO_2_ mineral trapping (Zhao et al., 2014). Soil microbes facilitate well-established conditions for the growth of plants by facilitating the weathering of rocks.

Currently, the regulatory function of microbial communities on ecosystem function has been extensively investigated; however, drivers of subsurface microbial communities remain largely unexplored. Microbial communities are regulated by soil nutrients. For instance, increased carbon input promotes soil microbial processes and plant growth. However, adding carbon to soil significantly increases soil microbial biomass and activity, but it has limited implications on the soil microbial community structure (Ma et al., 2012). Compared with soil conditioners, microbial addition to existing soil microbiota alters the structure of soil microbiota. Besides, adding soil microbes plays a vital role in the re-construction and regulation of subsurface microbial communities, significantly improving the abundance of beneficial soil microbes (Dong et al., 2019). Adding soil microbes also shortens the time for microbial agents to improve soil quality compared to compost (Zhuang et al., 2021). Thus, phytoremediation of exposed high steep rock walls progresses in the correct direction.

In phytoremediation, leguminous tree and shrub species are widely used due to their stress resistance, rapid regeneration, and the ability to restore a degraded environment (Mahmud et al., 2020). These plants reestablish degraded ecological functions and soil characteristics and positively affect the subsurface microbial community (Hu et al., 2015). In this study, the high steep slope of Yueyang Avenue in Hunan, China, was selected as an experimental site. Soil microbes in minerals were screened through rock weathering experiments. We studied the effects of microbial bacterial agents on the growth of legumes and soil physicochemical properties in combination with potting experiments. Besides, we also studied the intrinsic mechanism of bacteria-mediated plant growth promotion, intending to provide plant-growth promoting bacterial agents for efficient ecological restoration of challenging sites, such as high steep bare rock slopes. In a previous study, we proposed a hypothesis of “the imposed effect for promoting leguminous plant growth,” including mineral weathering, nodule growth promotion, and beneficial microbial regulation, and tested by fungi Penicillium simplicissimum NL-Z1 (Zhuang et al., 2021). In this study, we focused on the effects of microbial bacterial agents on the growth of legumes and soil physicochemical properties in combination with potting experiments.

## Materials and methods

### Sample source

The soil samples for screening microbial monocultures were obtained from the soil of the legume *Robinia pseudoacacia* from the high steep bare rock slopes of Yueyang Avenue, Yueyang City, Hunan Province, China. These soil samples were sieved through 0.075 mm for physical and chemical properties. In addition, local rock samples were collected from the same site and transferred to the laboratory. These rock samples were cleaned, crushed, and ground for compositional analysis of rock samples and subsequent weathering experiments.

### Screening and rock weathering experiments

#### Culture medium

(a) Phosphorus solubilizing strain isolation medium: 0.3 g sodium chloride, 0.3 g potassium chloride, 0.5 g ammonium sulfate, 0.3 g magnesium sulfate heptahydrate, 0.03 g ferrous sulfate heptahydrate, 0.3 g manganese sulfate tetrahydrate, 5.0 g calcium phosphate, 10 g sucrose, 15-20 g agar, deionized water 1,000 mL, and pH 7.0-7.5.
(b) Beef paste peptone medium: 3 g beef (dip) paste, 10 g peptone, 5 g sodium chloride, 20 g agar, 1,000 mL deionized water, and pH 7.0-7.2.
(c) Monkina organic phosphorus medium: 10 g glucose, 0.5 g ammonium sulfate, 0.3 g sodium chloride, 0.3 g potassium chloride, 0.3 g magnesium sulfate heptahydrate, 0.03 g ferrous sulfate heptahydrate, 0.03 g manganese sulfate, 5.0 g calcium carbonate, 0.3 g lecithin, 20 g agar, deionized water 1,000 mL, and pH 7.0-7.5.
(d) Monkina inorganic phosphorus medium: 10 g glucose, 0.5 g ammonium sulfate, 0.3 g sodium chloride, 0.3 g potassium chloride, 0.3 g magnesium sulfate heptahydrate, 0.03 g ferrous sulfate heptahydrate, 0.03 g manganese sulfate, 5.0 g tricalcium phosphate, 20 g agar, 1000 mL deionized water, and pH 7.0-7.5.
(e) Modified Monkina medium: In this medium, phosphorus-containing components mentioned in mediums 3 and 4 were replaced with rock samples.
(f) LB liquid medium: 10 g peptone, yeast dip powder 5 g, sodium chloride 5 g, deionized water 1,000 mL, and pH 7.2.

#### Isolation and purification of strains

A tenfold serial dilution of soil samples were smeared on NA and PDA to isolate bacteria and fungi, respectively. Three replicates of each diluent were cultured at 28°C for 3 days in a constant-temperature incubator. Single strains were obtained through three rounds of purification, and the isolated strains were stored at 4°C.

##### Screening of phosphate-dissolving bacteria

The screening of phosphate solubilizing microbes were carried out on Monkina agar at 28°C following spot inoculation; three replicates were prepared for each strain. The plates were treated with organic and inorganic phosphorus for 5 and 7 days, respectively, at 28°C in an incubator. The results showed that the transparent circle in the medium was due to the phosphate-dissolving bacteria. The diameter of the colony (d) and of the transparent circle (D) were measured, and D/d was calculated to preliminarily evaluate the ability of the strain to dissolve phosphate, five strains of phosphate solubilizing microbes were selected for weathering experiment.

##### Rock weathering experiments

In this study, single microbial colonies were isolated and cultured from rocks. To isolate microbes with mineral weathering ability and understand phosphorus weathering process, screening of soil was carried out. To verify if the rock can be dissolved, five different single microbial colonies with good phosphorus dissolution effect were selected for rock weathering dissolution analysis to explore the rock weathering effect of these microbes. After culturing the microbes in nutrient agar media, these five single colonies were added to a 100 mL conical flask containing 30 mL LB medium and oscillated at 30°C and 180 RPM for three days. 1 mL bacterial culture was added to a 30 mL modified Monkina broth in an Erlenmeyer flask containing 1.5 g rock particles instead of phosphorus in the modified Monkina agar and incubated at 30°C and 180 RPM for ten days. At different time intervals (4, 7, and 10 days), samples (*n* = 3) after centrifugation (8000 RPM, 10 min) were collected from the inoculated group (X-4, X-8, X-11, X-14, X-45) and the control group. The resulting 5 mL of supernatant was added to 0.2 μm pour filtrate and centrifuged to determine the pH value, phosphorus (AP), potassium (k), calcium, and magnesium content. 100 mL Erlenmeyer flask containing 30 mL LB media was incubated at 30°C and 180 RPM for three days. Then, 1 mL broth of each five microbial strains was inoculated into the 30 mL modified Monkina broth media containing 1.5 g rock particles to replace the phosphorus component of Monkina agar in an Erlenmeyer flask and incubated at 30°C and 180 RPM for 10 days. Samples (*n* = 3) from inoculated groups (X-4, X-8, X-11, X-14, X-45) and the control group were collected on day ten and centrifuged at 8,000 RPM for 10 min. 5 mL of supernatant was filtered using a 0.2 μM membrane filter for the determination of pH, available phosphorus (AP), potassium (K), calcium (Ca), and magnesium (Mg). pH was determined using the Mettler toledo pH meter. AP was determined using the molybdenum-antimony anti-colorimetric method. K, Ca, and Mg was determined using the atomic absorption spectrometer. The shape and size of the rock particles were measured during the last sampling.

### Potted plant experiment

#### Experimental environment

The experiment was conducted in a greenhouse at the teaching base of Nanjing Forestry University in Baima, with 65% air humidity, 450 ppm CO_2_ concentration, 28°C and a maximum photosynthetic radiation of 1,850 μmol/(m^2^⋅s). The soil was taken from the field trial fields below 20 cm at the Baima site and brought back to the laboratory to remove any gravel, dead material and roots, etc. The soil was sterilized in an autoclave at 121°C for 30 min to ensure sterility. Plastic pots with an inner diameter of 9 cm, an outer diameter of 13 cm and a height of 15 cm were used for planting *I. pseudotinctoria.*

#### Bacterial agent preparation

To produce bacterial X-45 inoculum, isolates were grown to a certain colony size on NA at 28°C and then inoculated into liquid medium at 30°C and 180 RPM for three days. The OD 600 of this media was measured by UV spectrophotometer, and the OD 600 value was adjusted to 0.8∼1.2. Later, the media was sealed and stored in 4°C refrigerators.

#### Experimental design

The bacterial strain X-45, which was effective in the weathering experiments, was selected for potting experiments to study the growth-promoting ability of this strain. The experiment was divided into a control group and an experimental group. Leguminosae *I. pseudotinctoria* were used as experimental plants. The pots were divided into two groups (CK and X-45, 3 replicates for each group) and placed in a greenhouse. Three young shoots of *I. pseudotinctoria* were planted in each pot and screened after one month of growth, keeping one healthy seedling in each pot (the same growth from pot to pot). For inoculation, the stored bacterial solution was diluted 100 times, 60 mL of diluted bacterial solution was added to each pot, and three replicates were set for each group, while an equal amount of sterile culture solution was added to the control group. Three months later, the plants were collected and the indexes were measured.

#### Determination of potted plant indicators

For plants: Vernier caliper and tape were used to measure the ground diameter and height of seedlings. Measuring the quantity and quality of root nodules. The leaf area was measured using a root scanner (10 upper, middle, and lower leaves of each pot were selected to measure the leaf area). Plants were sampled and dried, and the above-ground and underground biomass of the plants were measured, respectively.

For potted soils: The hydrolytic nitrogen of the soil was determined by the alkaline diffusion method. The effective phosphorus of the soil was determined using the acid soluble-molybdenum antimony anti-colorimetric method, and the pH was determined by a Mettler Toledo pH meter (water to soil ratio of 5:1).

### Microbial strain identification and macro-genome sequencing

#### Identification of the physiological and biochemical characteristics

Physiological and biochemical characteristics, including the morphology of the bacteria, Gram test, amylase hydrolysis test, V.P test, methyl red test, hydrogen sulfide production test, gelatin liquefaction test, milk coagulation and peptonization test, and the nitrate reduction test *Common Identification Method of Bacteria and Characteristics and Yeast Identification Manual*.

#### 16S rDNA identification of the gene sequence

The bacterial X-45 strain was identified using morphological characterization and 16s rRNA analysis. Molecular-level identification was performed with the support of Shanghai Jinyu Medical Laboratory Co., Ltd., The collected and collated rhizobial inter-rhizosphere soil samples were sent to Shanghai Mayobio Biomedical Technology Co., Ltd., for sequencing on the Illumina Miseq platform. The sequencing primer was 515F_907R.

#### Identification

As shown in Table 1, strain X-45 is a kind of bacteria producing bright red pigment, which is motile, convex, irregular on edge, unable to ferment sugars, and positive in Starch hydrolysis, indole, methyl red, V.P., citrate and hydrogen sulfide tests.

**TABLE 1:** Morphological physiological and biochemical characteristics of strain X-45.

Project	Characteristics	Project	Characteristics
Colony characteristics	Edge irregularity, Red, Bulge	Starch hydrolysis	+
Gram stain	Red, Negative	Indole test	+
Morphology	Short rod	Methyl red test	+
Motility	+	V.P. test	+
Glucose Fermentation (gas/acid)	−/+	Citrate test	+
Lactose Fermentation (gas/acid)	−/−	Hydrogen sulfide test	+

“+” means that the test result is positive; “−” means that the test result is negative.

The 16S rDNA sequence of strain X-45 was compared and analyzed by using MEGA (version 6.0) software. As shown in the Figure 5, the phylogenetic tree was constructed. The sequence result was similar to that of *Serratia marcescens* by BLAST, and the similarity was 99.79%. Combining the previously mentioned results, the strain X-45 was confirmed as *Serratia marcescens* N1.14.

**FIGURE 1:**
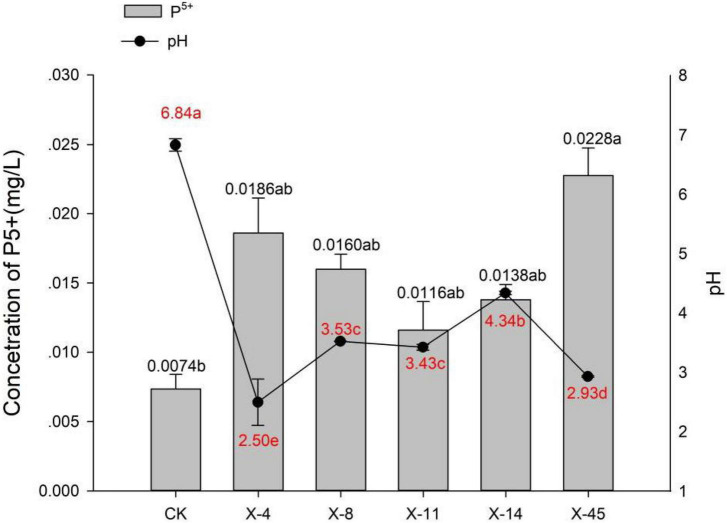
Release of active phosphorus and pH changes.

**FIGURE 2:**
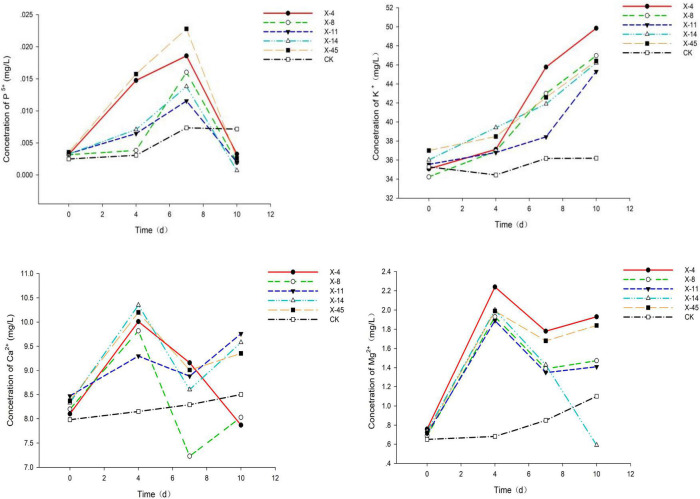
Phosphorus, potassium, calcium, and magnesium release from the fermentation broth of five strains of bacteria.

**FIGURE 3:**
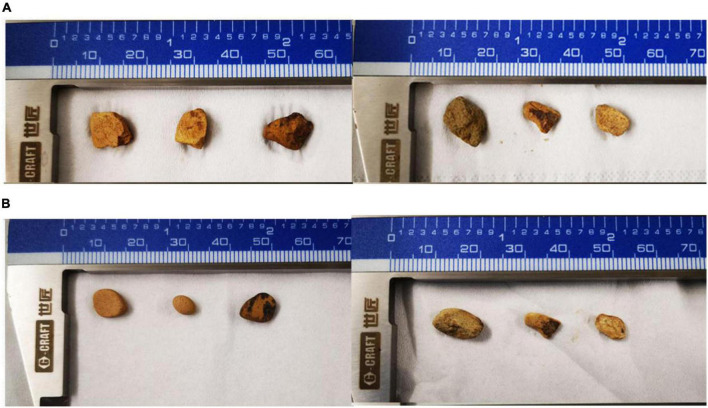
The dissolution of the rock after 10 days by *P. simplicissimum*. **(A,B)** Means three replicates in the X-45 treatment group before and after the experiment, respectively.

**FIGURE 4:**
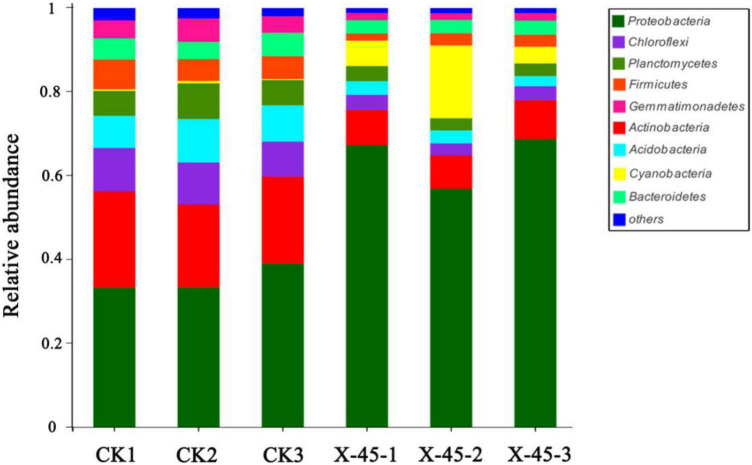
Relative abundance of bacterial communities at phylum.

**FIGURE 5:**
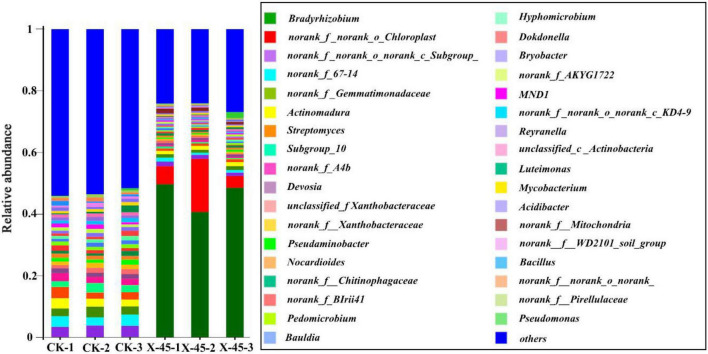
Relative abundance of bacterial communities at genus level.

The nucleotide sequence of X-45 and the potted soil 16S sequence have been uploaded to the NCBI database, the registration numbers are MT645673^1^ and PRJNA645742^2^, respectively.

### Statistical analysis

The bacterial sequences were submitted to the GenBank database^3^. Raw sequence data and sequencing quality files were obtained as FASTA files, and file access was used for processing and analysis through Mothur software as described previously by Schloss (Hakim et al., 2019). To account for potted soil microbial diversity as well as the abundance of dominant species, α-diversity indices (Chao1, Simpson, and Shannon indices) and relative abundance were quantified using OTU richness. In addition, the analysis detected the relationship between environmental factors, samples, and flora using the ggplot2 package; the ggplot2 package was set as the default parameter in R, and the vegan package was set as the default parameter in R for environmental association analysis. Plant indicators and soil physical and chemical property indicators were analyzed using spss software. Analysis of function difference between groups was calculated by Welch’s *t*-test (Oksanen et al., 2011). Analysis of variance (ANOVA) was performed on plant biomass and soil physicochemical property data (Systat Inc., Evanston, IL, USA), using Student Newman-Keuls significant difference (*p* < *0.05*) to distinguish treatments from controls (Manly, 2018).

## Results and analysis

### Screening of phosphate-dissolving bacteria

According to the results of the transparent phosphate-dissolving circle on the Monkina organic phosphorus and inorganic phosphorus culture plates, a strain with the ability of phosphate-dissolving was screened out. And the diameter of the transparent dissolved phosphate rings was D.

The colony diameter is represented by d, and D/d indicates the phosphate-dissolving effect of the strain: the larger the proportion, the better the effect of the dissolving phosphate. A total of 58 strains of bacteria were isolated from the soil samples in this experiment. As shown in Table 2, 21 strains were screened, of which 15 were organo phosphate-dissolving bacteria, and 15 were inorganic phosphate-dissolving bacteria. Nine strains could degrade both organo phosphorus and inorganic phosphorus. Finally, five strains with good effect were selected for further study, namely X-4, X-8, X-11, X-14 and X-45.

**TABLE 2:** Primary effect of phosphate-dissolving bacteria.

Name	D/d(organic)	D/d(inorganic)	Name	D/d(organic)	D/d(inorganic)
X-4	3.61a	2.13c	X-35	—	1.27f
X-8	—	2.60bc	X-38	2.41bc	1.93c
X-11	3.06ab	4.54a	X-42	—	1.57d
X-14	3.47a	2.81b	X-43	2.83b	—
X-17	2.22c	—	X-44	1.42e	—
X-19	2.09cd	2.60bc	X-45	3.95a	1.67d
X-25	2.49b	1.53d	X-48	2.25c	—
X-27	2.50b	2.00c	X-53	1.79d	—
X-30	—	1.69d	X-55	2.22c	1.37f
X-33	—	1.50d	X-58	2.17c	—
X-34	—	1.44df			

D/d indicates the phosphate-dissolving effect of the strain: the larger the proportion, the better the effect of the dissolving phosphorus. Different letters in the columns indicate significant differences (*P* < 0.05).

### Weathering effect of microbial strain X-45

Based on the results of the analysis of the rocks, the main components of the rock samples are shown in Table 3.

**TABLE 3:** Composition of rock sample.

Species	K_2_O	Na_2_O	CaO	MgO	P_2_O_5_	Fe_2_O_3_	Al_2_O_3_	MnO
Concentration/percentage	3.71	1.39	0.21	1.28	0.11	6.81	15.21	0.04

Five isolated bacteria were compared based on the release of effective phosphorus and the pH variation. With a concentration of 0.0228 mg/L, effective peak phosphorus released from microbial strain X-45 was the highest, which was 3.08 X higher than the control group (Figure 1). The pH of the fermentation broth was also determined. The pH of the group treated with X-45 decreased significantly relative to the blank control group. In addition, the X-45 microbial strain showed a greater ability to release each element into fermentation broth (Figure 2), and its peak release for potassium, calcium, and magnesium increased by 25.43, 11.71, and 155.56%, respectively, compared to the control. Overall, the release of the P, K, Ca, and Mg in the rock particles by each strain initially showed an upward trend followed by a downward trend. When the strains were in their growth phase, the concentration of the elements in the fermentation broth increased continuously. However, the strains in the growth phase would eventually consume large amounts of nutrient elements in the fermentation broth; moreover, the fermentation space is limited. Thus, the element dissolution rate is lower than the utilization rate, resulting in a downward trend. At the end of the experiment, we found that the rock particles became significantly smaller, and their morphology changed dramatically (Figure 3). The results showed that microbial strain X-45 effectively released each element and changed rock morphology significantly, indicating that X-45 can effectively promote rock dissolution.

### Growth promotion of X-45

After treatment with microbial strain X-45, all above-ground indices of *I. pseudotinctoria* seedlings were higher than the sterile treatment group. With a significant increase of 22.7%, the above-ground biomass of the treated groups was 7.33 g. In addition, the ground diameter increased significantly by 23.52%, seedling height by 18.91%, and leaf area by 30.48% to the average values of 5.62 mm, 65 cm, and 6.55 cm^2^ (*P* < *0.05*), respectively.

In this study, the root nodules of the experimental plants were analyzed. The average number of root nodules formed in the sterile treatment group was about 8 cm, and the total mass of root nodules was about 0.19 g. The average number of root nodules formed in the seedlings treated with microbial strain X-45 was 86, and the total mass of roots of the seedlings treated with microbial strain X-45 was about 0.42 g. Compared with the control group, the number of root nodules and total mass increased significantly by 11.21 X and 121.05% in the seedlings treated with microbial strain X-45, respectively. In addition, the root biomass, root surface area, and root volume of the sterile treatment group were 1.2 g, 232.26 cm^2^, and 1.71 cm^3^, respectively. The root biomass of the seedlings treated with microbial strain X-45 increased by 45% to 1.74 g (*P* < *0.05*), root surface area increased significantly by 13.91% to 264.57 cm^2^, and root volume increased significantly by 53.8% (*P* < *0.05*) to 2.63 cm^3^ (P < 0.05).

In addition, the physical and chemical properties of *I. pseudotinctoria* potted soil also changed. With a significant increase of 22.76% (*P* < *0.05*) and 19.95%, concentrations of available phosphorus and hydrolyzed nitrogen of the potted soil treated with microbial strain X-45 were 3.02 mg/kg and 248.5 mg/kg, respectively. The potted soil was acidified to a certain extent, and the pH decreased from 7.06 to 6.89. Pot experiment further confirmed that microbial strain X-45 could convert phosphorus and nitrogen in soil into forms directly absorbed and utilized by plants to promote plant growth and create an environment conducive to the growth of *I. pseudotinctoria* plants. The growth-promoting effect of X-45 microbial strain on *I. pseudotinctoria* was further verified (Table 4).

**TABLE 4:** Comparison of physicochemical properties of soil plants.

Groups (sample)	Plant (underground)			Plant (aboveground)				Soil (potted,mg/kg)		
	Total nodule weight(g)	Dry weight(g)	Root volume(cm^3^)	Dry weight(g)	Ground diameter(mm)	Plant height(cm)	Average leaf area(cm^2^)	AP	HN	pH
CK	0.19 ± 0.06b	1.20 ± 0.07b	1.71 ± 0.02a	5.74 ± 0.07b	4.55 ± 0.39b	54.67 ± 2.52b	5.02 ± 0.30b	2.46 ± 0.36b	207.17 ± 3.21a	7.06 ± 0.06a
X-45	0.42 ± 0.07a	1.74 ± 0.11a	2.63 ± 0.11a	7.33 ± 0.36a	5.62 ± 0.16a	65.00 ± 5.57a	6.55 ± 0.21a	3.02 ± 0.33a	248.50 ± 4.58a	6.89 ± 0.10a

The values “a, b” represent the standard deviation. Different letters represent significant differences. Abbreviations are HN: Hydrolyzable nitrogen; AP: available phosphorus.

### Effect of X-45 microbial strain inoculation on soil microbiota

**FIGURE 6:**
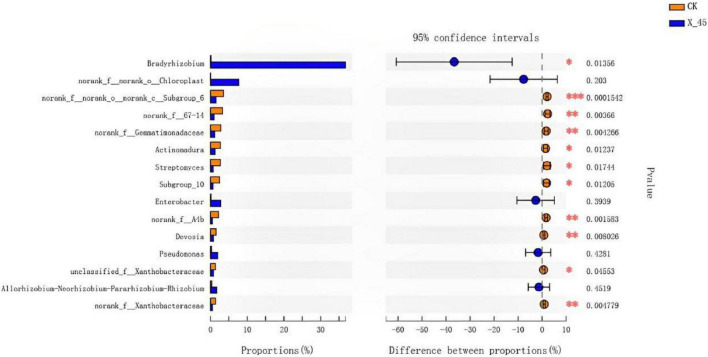
Student’s *t*-test bar plot on genus level with 95% confidence intervals.

The microbial diversity in potted soil was detected using high-throughput sequencing. The microbial community composition of potted soil was also analyzed. There was no difference in microbial community composition at the phylum level between the control group and the potted soil treated with the microbial strain X-45. However, a remarkable difference was observed in the relative abundance of microorganisms between these two groups. The Proteobacteria abundance increased from 35% to 64% (Figure 4). *Bradyrhizobium* (circa 46%) was found to be the dominant genus in the X-45 treated group, while the relative abundance of *Bradyrhizobium* in the control group was less than 0.5% (Figure 5). The student’s t-test was used to test the significance of species differences at the genus level (Figure 6). *Bradyrhizobium* was found to be significantly different between the two treatment groups (*P* < *0.05*).

### Redundancy analysis

**FIGURE 7:**
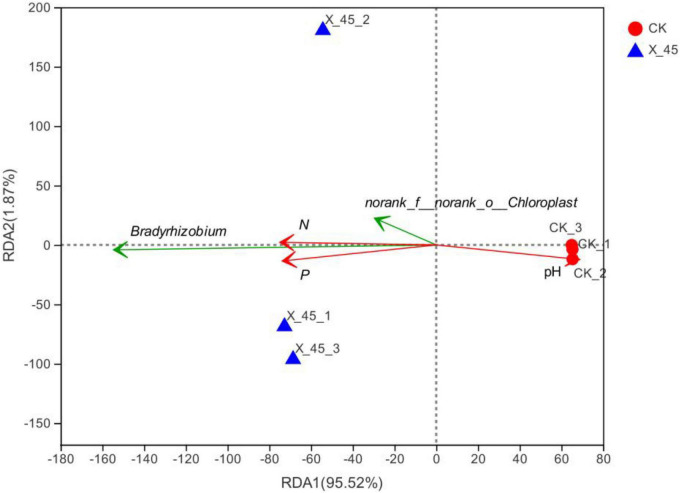
Bacterial community redundancy analysis (RDA).

Redundancy analysis (RDA) was conducted at the genus level to reflect the relationship among environmental factors, flora, and samples (Figure 7; axis 1 = 95.52%, axis 2 = 1.87%). This analysis showed that community distribution was significantly and positively correlated with available phosphorus (*r*^2^ = 0.91, *P* < 0.01), significantly and positively with hydrolyzed nitrogen (*r*^2^ = 0.99, *P* < 0.05), and negatively with pH (*r*^2^ = 0.97, *P* < 0.05). Out of the identified genera, *Bradyrhizobium* showed the strongest correlation with various environmental factors (Figure 7).

## Discussion

The growth of plants is mainly dependent on the soil. However, the poor conditions of high and steep exposed slopes restrict the phytoremediation process. This study aimed to screen and isolate the microorganisms that could improve the soil environment and thus the efficiency of phytoremediation. In this study, the Mongina organic (inorganic) phosphorus medium screening method was used to screen phosphate-dissolving bacteria. Research has shown that the transparent circle on the Monkina plate and the available phosphate concentration in the fermentation broth can only preliminarily explain the phosphate dissolving ability of phosphate-dissolving bacteria and cannot further reliably evaluate their phosphate dissolving effect (Calvaruso et al., 2013). Therefore, in this study, rock particles were used instead of phosphate in Mongina culture medium for dissolution experiments, and the ability of the phosphate-dissolving bacteria was determined by analyzing the changes of phosphate in the fermentation broth. A considerable release of K, Ca, Mg, and active phosphorus by soil microbes in X-45 treated soil was observed through rock weathering experiments. In addition to the decrease in pH (Wang et al., 2018), previous studies have shown that microbes could secrete H + to dissolve minerals, validating the acidification effect of microbes (Azaiez et al., 2018). In this study, the rock weathering ability of microbial strain X-45 accelerated the soil formation process, improved the soil quality, and provided mineral nutrients required for plant growth.

Symbiotic nitrogen fixation of rhizobia in legume nodule injects about 40 million tons of nitrogen into the agricultural system every year (Udvardi and Poole, 2013), increasing plant nutrient content, improving soil health reclamation, and reducing synthetic nitrogen fertilizer application in agriculture (Mahmud et al., 2020). In the pot experiment, we observed that the number of root nodules of *I. pseudotinctoria* inoculated with microbial strain X-45 was 11.21X that of the control group. The total weight of root nodules increased by 121.05%, and hydrolyzed nitrogen content in pot soil increased by 19.95%. With increasing nitrogen content, X-45 microbial strain promoted the nodulation and nitrogen fixation in *I. pseudotinctoria* (Mu et al., 2018). Thus, the symbiotic nitrogen fixation ability of root nodules and host plants was effectively brought into play.

In this study, the microbial strain X-45 converted soil phosphorus and nitrogen to a form that could be directly absorbed and used by the plant. Also, a significant improvement was noticed in the hydrolytic nitrogen and effective phosphorus in the potted soil treated with microbial strain X-45. This validated that in this study, phosphorus-solubilizing bacteria in the experimental soil slowed down the fixation of effective phosphorus and promoted the growth of *I. pseudotinctoria* (Chungopast et al., 2021). The isolated microbial strain X-45 belongs to the genus Serratia. Previous studies have shown that it could transform insoluble phosphorus into accessible phosphorus and be used as an inoculant to increase phosphorus uptake by plants (Srinivasan et al., 2012). The above analysis proved that X-45, a phosphorus solubilizing bacterium, released phosphorus effectively. Besides, it improved soil quality and promoted plant growth. Thus, microbial strain X-45 could be used as a functional strain for phosphorus solubilization and microbial fertilizer.

Microbial agents could change soil microbial community structure. In this study, we observed that the application of microbial agent X-45 directly increased the relative abundance of *Proteobacteria*. *Serratia marcescens* (the original genus of X-45) was not found in the potted soil; however, the dominant genus was found to be *Bradyrhizobium*, which belongs to *Proteobacteria* just like microbial strain X-45. *Bradyrhizobium*, a type of rhizobium that grows in the roots of legumes, has nitrogen fixation ability (Hungria et al., 2015). Thus, it can improve the nodulation rate and nodulation amount (Masciarelli et al., 2014), increase nutrient content and dry matter accumulation (Gough et al., 2021), improve soil quality, and decrease soil degradation (Li et al., 2018). Previous studies have shown that soil microbes play an important role in the growth and health of plants. Relevant studies have shown that the relative abundance of *Proteobacteria* in healthy soil is higher than in soil infected with bacterial wilt (Yuan et al., 2020). A higher abundance of beneficial microorganisms improves soil quality and thus promotes a lower incidence of morbidity, higher nutrient content, and soil enzyme activity (Wang et al., 2017). In addition, according to the RDA analysis of environmental factors and bacterial communities, such bacterial communities as *Bradyrhizobium* are significantly positively correlated with available phosphate and hydrolyzed nitrogen, indicating that X-45 indirectly improves the release of nutrients by promoting the increase in the abundance of *Bradyrhizobium*. Therefore, X-45 can be used as a rhizobium growth promoter, and it is potentially important when planting legumes in poor soil areas for slopes protection.

In this study, we observed that X-45 microbial strain inoculant could rapidly shorten the soil improvement cycle compared with organic fertilizer. Previous studies have shown that after 35 years of field experiments with soybean compost, the abundance of beneficial microorganisms in the soil increased significantly, from less than 1 to 40% of the fungal species (Li et al., 2018). Compared with this study, experimental results of the current study showed that three months after inoculation with microbial strain X-45, the abundance of *Aspergillus* in the test group increased from 35 to 64%, and the abundance of *Bradyrhizobium* increased from less than 1 to 42%. This suggested that X-45 can rapidly shorten the time span of the improvement cycle of soil beneficial microbial populations compared to organic fertilizer application.

In a previous study, the hypothesis of “the imposed effect for promoting leguminous plant growth,” including mineral weathering, nodule growth promotion, and beneficial microbial regulation, was based on the role played by fungi *Penicillium simplicissimum* NL-Z1 (Zhuang et al., 2021). This study also confirmed the hypothesis of the superposition effect of legume promotion via bacteria. According to previous studies, the inoculation of beneficial microorganisms, such as fungi and bacteria, on legume plates may differ significantly from harmful microorganisms.

## Conclusion

In this study, the highly efficient phosphorus-dissolving lithotrophic bacterium X-45 was isolated from soil samples of Yueyang Avenue, Yueyang City, Hunan Province, China, by conventional methods and rock weathering experiments. The pot experiment showed that microbial strain X-45 increased the legume’s symbiotic nitrogen fixation effect and promoted plant growth. The microbial analysis at the genus level showed that microbial strain X-45 substantially enhanced the growth of *Bradyrhizobium*. The ability of *Bradyrhizobium* to improve the physicochemical properties of the soil was verified using RDA analysis. Based on the above analysis, we concluded that the beneficial changes in soil flora of *I. pseudotinctoria* potted plants were due to bacterial agent X-45. Besides, the addition of X-45 for soil improvement, combined with symbiotic nitrogen fixation by legumes, indirectly regulated the abundance of beneficial soil microbes and improved soil quality. The interconnection of mineral weathering, rhizome promotion, and microbial regulation significantly promoted the growth of *I. pseudotinctoria* by improving soil quality, which is of great importance for slope restoration.

## Data availability statement

The datasets presented in this study can be found in online repositories. The names of the repository/repositories and accession number(s) can be found below: The nucleotide sequence of X-45 and the potted soil 16S sequence have been uploaded to the NCBI database, the registration numbers are MT645673 and SRP271689, respectively.

## Author contributions

JXZ and JYZ: conceptualization, designed the experiments, performed the experiments, analyzed the data, and wrote the draft. JXZ and JL contributed clinical advices and review and editing the manuscript. JXZ and CL designed the research study, wrote the manuscript, and supervision. All authors read and agreed to the published version of the manuscript.

## Footnotes

1
https://www.ncbi.nlm.nih.gov/nuccore/MT645673

2
https://dataview.ncbi.nlm.nih.gov/objects?linked_to_id=PRJNA645742&archive=sra

3
http://www.ncbi.nlm.nih.gov
