# Supplementary material for: Study of the effect of bacterial-mediated legume plant growth using bacterial strain Serratia marcescens N1.14 X-45
Source: Front Microbiol. 2022 Oct 24;13:988692. doi: 10.3389/fmicb.2022.988692 (PMC9638080; doi:10.3389/fmicb.2022.988692)
Supplement: Supplementary file 1 [file Data_Sheet_1.pdf]

Supplementary Tables. Effects of X-45 on plant and soil

Groups (Sample)	Soil (potted)			Plant (above ground)			Plant (underground)		
	HN (mg/kg)	AP (mg/kg)	pH	Ground Diameter (mm)	Plant Height (cm)	Average Leaf area (cm^2)	Number of nodules	Total nodules weight(g)	Dry weight(g)
CK	203.50	2.461	7.12	4.64	57	4.71	4	0.202	1.28
	209.50	2.497	7.01	4.12	52	5.30	12	0.237	1.19
	208.50	2.426	7.05	4.88	55	5.06	7	0.122	1.14
X-45	252.50	3.351	6.91	5.78	71	6.76	96	0.466	1.85
	243.50	2.701	6.93	5.46	60	6.34	86	0.419	1.74
	249.50	3.021	6.84	5.62	64	6.55	76	0.372	1.63
